# Adoption of sustainable fuels and renewable energy in logistics: Examining the role of stakeholder theory in supply chain strategy

**DOI:** 10.1371/journal.pone.0320507

**Published:** 2025-03-25

**Authors:** Chukwudi Christian Ifekanandu, Chiemelie Benneth Iloka, Grace Ifeoma Anukwe, Jude Obinna Eze, Innocent Vitus Uwakwe, Samuel Asagba

**Affiliations:** 1 University of Nigeria Business School Enugu, University of Nigeria Enugu Campus, Enugu, Enugu State, Nigeria; 2 Department of Marketing, Enugu State University of Science and Technology (ESUT), Agbani Main Campus, Enugu, Enugu State, Nigeria; 3 Department of Marketing, UNN Business School Enugu, University of Nigeria Enugu Campus, Enugu, Enugu State, Nigeria; 4 Department of Marketing, Delta State University of Science and Technology, Ozoro, Delta State, Nigeria; University of Central Punjab, PAKISTAN

## Abstract

In contemporary society, sustainability is a key issue that borders on overall human existence, and it has been rigorously argued that extensive efforts are needed to ensure the survival of the human race. Such efforts include corporate bodies changing the ways they operate to ensure that their activities do not yield negative impacts on the environment and the human race in general. In view of this, this research was conducted to assess the influence of stakeholder theory (consumers’ demand, suppliers’ collaboration, and regulatory pressure) on the decision of companies in the logistics sector to adopt sustainable fuel and renewable energy in their operations. The study also sought to assess the mediating role of organisational commitment. To this end, 294 responses were gathered across companies in three states in Nigeria. Gathered data were analysed using SPSS and SmartPLS software. Findings indicate that stakeholder theory (consumers’ demand, suppliers’ collaboration, and regulatory pressure) yields significant influence on the adoption of sustainability measures (sustainable fuel and renewable energy) in logistics companies. Additionally, organisational commitment was found to mediate this relationship. Therefore, it was concluded that stakeholders should put pressure on logistics companies to adopt sustainability measures because they yield significant influence on that decision.

## 1. Introduction

In the past few decades, there has been an increase in the number of studies that focus on assessing how global warming or other rampant increases in greenhouse gases negatively affect the daily lives of man [1]. These issues are man-made conditions that have severely affected the ecosystem in a harmful way, with clear instances of negative impacts on economic growth and human health [2]. The most frequently mentioned areas that are being affected are food production, environmental conditions, and access to water, and these affected areas have caused great damage to the sustainability of the human race [1]. Therefore, it is projected that in the near future, these issues will likely get worse. Evidence from the medical sector has shown that there is an increase in the level of dengue, diarrhoea, and malaria, all due to issues linked to climate change. Aside from the numerous respiratory disorders, there are increasing concerns about cardiovascular health, especially as the heatwaves and air pollution continue to increase as a result of industrial purposes, of which one of them is the generation of power [3]. On a similar note, the agricultural sector has been severely affected by alteration of the ozone layers and the continued rise in sea levels, with indirect effects on human health in the area of inadequate nutrition. Therefore, as day passes by, it is becoming clear that there is a need for both individuals and corporations to adopt renewable sources of energy as opposed to relying on conventional sources (fossil fuel); this is not only based on the fact that coal and natural gases are being depleted as fuel but also avert further damage to the environment, as such damages can further affect health and make it more difficult for the future generation to survive [4].

In the logistics sector, the importance of optimising operations to align with these demands for renewable sources of energy is also clearly evidenced. This is commonly known as green logistics. As stated by Vienažindienė et al. [5], the application of green logistics in the business setting is a vital prerequisite for reducing the negative impacts that logistics yield on the environment. Implementing green practices in logistics operations has been described as the only solution for controlling climate change, air pollution, and global warming issues [5]. In their study, Aldakhil et al. [6] were able to demonstrate that green practices in logistics operations yield positive influence on sustainable environmental and economic growth. Therefore, the possibility of attaining the objectives of environmental and economic growth is said to be dependent on the effectiveness and visibility of green practices that logistics companies implement in their operations [7]. On the same note, Karaman et al. [8] and Seroka-Stolka and Ociepa-Kubicka [9] stated that adopting green logistics practices helps to promote a country’s circular economy and overall economic development. The activities of green logistics that help in stimulating the circular economy of enterprises include the use of environmentally friendly green packaging, storage, green transportation, and sustainable processing flows [9].

It has been emphasised in a significant number of scholarly works [10–12] that companies can sustain their development by making sure that they actively apply green logistics practices within their corporate activities: transportation, storage, and packaging. Furthermore, studies on the application of green logistics have been undertaken in countries like Germany, Italy, Great Britain [13], Sweden [14], Slovakia [15], China [16], Monaco 17, Lithuania [5], and Thailand [18]. These studies reveal wide differences in the variety of green logistics practices being deployed by companies. Findings from these studies show that the application of green logistics practices in countries can differ based on the duration of the company’s activities, the culture of the country and business, and the basket of logistics services available, among other factors [5]. As opined by Vienažindienė et al. [5], from the research point of view, the majority of the studies conducted about green logistics practices have actually focused on the manufacturing sector, with limited attention being paid to the logistics and transportation service providers. On the same note, there is a high level of variability as to how these green logistics practices are being implemented by the logistics companies as well as the factors that enhance or hinder their overall application. Therefore, while the academic world has played a significant role in analysing green logistics practices and how they are implemented, there is a significant gap in research designed to construct practical frameworks for implementation of green logistics practices for sustainable development in the logistics sectors.

The above discussions show that majority of the studies have focused on identifying the benefits that come with sustainable logistics practices, neglecting how such outcome can be achieved. In their study, Kuwornu et al. [19] highlighted the importance of understanding the internal and external practices that influence sustainable supply chain management, especially as there are limited researches within that context. Although Hmouda et al. [20] recognized the need to assess factors that influence sustainable practices in the logistics sector, their study primarily focused on scope related to sustainable chain practices in the energy sector. Therefore, there is an urgent need to address this existing gaps, especially as extant studies have highlighted the potential benefits understanding the role of sustainable energy and renewable fuels in the logistics sectors. Additionally, there is no extant study that have considered this topic from the dimension of stakeholders’ theory and this is an extension of this present study.

These gaps are what this present study aims to fill by considering adaptation of sustainable fuels and renewable energy in the logistics sector based on the implementation of stakeholder theory in the supply strategy. The major motivation for this study is the increased demand to create a sustainable environment and economy for the future generation, and it is believed that the logistics sector has a significant role to play in this regard for adopting sustainable measures in their operations. With increasing emphasis on sustainability, there is limited research on the effectiveness of green logistics practices, such as sustainable fuels and renewable energy infrastructure, and their integration into existing supply chain models. The findings from this study have both practical and academic significance. In the academic sense, it has been able to fill the void in terms of understanding how sustainable energy and renewable fuel can influence logistics operations based on the stakeholders’ theory, within the Nigerian context. An outcome that can be replicated in other sectors in the country, and other logistics sectors of different markets across the globe. In terms of practice, the findings have shown that sustainable practices in the logistics sector revolve around renewable energy and sustainable fuel, and its impact can be felt across the corporate and general world. In the corporate world, it will help companies to reduce operational costs and improve sustainable operations. On the same note, it will further enhance sustainability of our ecosystem, ensuring that the future generations are not denied the opportunity to meet their own needs. Therefore, the findings from this research, when further implemented, can provide vast positive results that can benefit the entire human race.

## 2. Theoretical framework

### 2.1. Stakeholder theory

Stakeholder, as a term, has its history from the Stanford Research Institute in 1963, but it has continued to evolve over the years, especially as it was featured in Freeman’s 1984 book, “Strategic Management: A Stakeholder Approach,” serving as the foundational work. It was posited by Freeman that the interests of stakeholders should be considered by companies instead of focussing solely on the shareholders, as this will ensure that the company is able to create value for everyone that is involved in its corporate process and attain success in the long run. This work was the formal introduction of stakeholder theory in the field of strategic management. Based on this theory, a stakeholder represents any entity (either individual or group) that can influence or is influenced by a mission-driven organisation (a company that has clearly defined objectives it has set out to accomplish). This was the foundation of stakeholder research, and it was further accompanied by other related studies that make significant contributions in the field.

The work of Freeman was expanded by Donaldson and Preston [21], and they argued that companies have the moral obligation to consider the interests of all stakeholders within their sphere and that effective management of stakeholders has the potential to bring about sustainable profitability. Their work highlighted three theoretical approaches for addressing the needs of stakeholders as: a) the descriptive approach – in which organizations are viewed as entities that comprise of different stakeholder groups with their varied interests (which is also known as the least comprehensive view, as it sets the stage for considering the claims or concerns of stakeholders), b) the instrumental approach – place emphasis on the important of stakeholder management because it brings about positive financial outcomes (that is, a progressive view which sets the stage for balancing the interests of stakeholders and financial interests of the company), and c) the normative approach – which considers the stakeholders to be the “end” and not the “means” to attaining financial outcomes (this is considered to be the most comprehensive view of stakeholders as it focuses on them).

In order to assess the influence of stakeholders, Mitchell et al. [22] did propose the attributes of legitimacy, power, and urgency, which, when viewed collectively, can function as the right indicator for identifying the vital management attention that should be accorded to the stakeholders. Other studies include the work of Jones and Wicks [23], which proposed a unified stakeholder theory that integrated different views and approaches. As acknowledged in their work, the field of stakeholder theory has become fragmented, as different authors have focused on different features of stakeholders. In order to address this issue, they went on to prescribe a unified theory of stakeholders that integrated the different perspectives: descriptive, instrumental, and normative. In this unified theory, the instrumental perspective focused on the strategic importance of stakeholders to the company, the normative perspective focused on ethical obligations that a company has towards its stakeholders, and the descriptive perspective assessed the empirical relationship between the interests of the company and those of the stakeholders. Through the integration of these perspectives, the intention of the author was to develop a more cohesive and comprehensive stakeholder theory. Phillips et al. [24] went further to classify what stakeholder theory is not, positing that stakeholder theory is not solely a normative theory of corporate social responsibility of a theory that is focused predominantly on business ethics. Instead, they pointed out that stakeholder theory is a foundational framework that is used to understand and manage the interests of different stakeholders, whose implications can further be extended to other areas like ethics, corporate social responsibility, and sustainability. In today’s context, stakeholders have become pivotal considerations across the different aspects of decision-making [25–29], and different strategies have been developed to ensure that the stakeholders are engaged effectively [30–35].

In view of the above discussions, it is theorised in this research that the interests of stakeholders yield influence on the decision of logistics companies to adopt sustainable fuels and renewable energy. This is because if the interests of the stakeholders are centred on the adoption of sustainable fuel and renewable energy, stakeholder theory posits that logistics companies should endeavour to meet these interests in order to create a sustainable competitive and performance edge.

## 3. Conceptual framework

**Fig 1:**
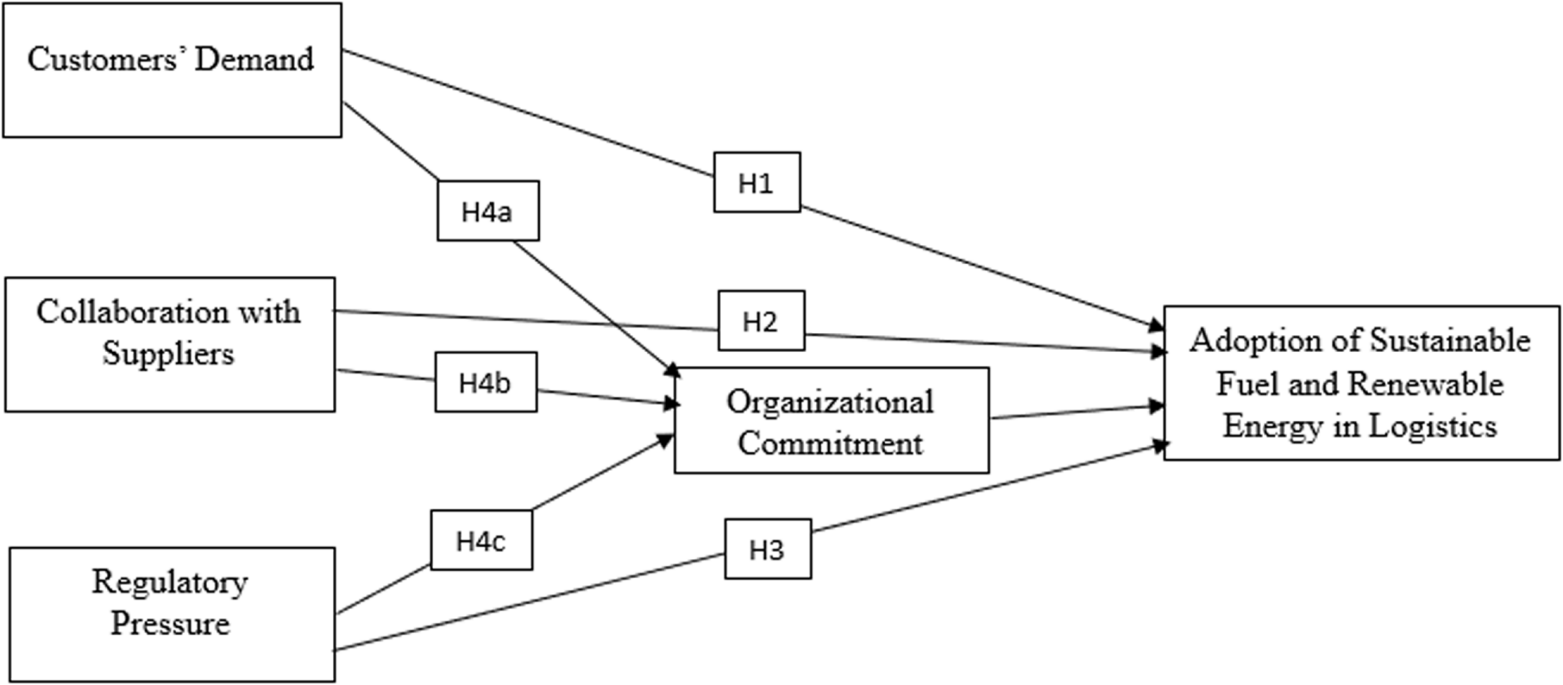
Conceptual framework. Source: Authors (2024).

The Fig 1 documents the relationship between the variables, conceptualized from the discussions above. It shows two layers of possible relationships, direct relationship between the independent variables (customers’ demand, collaboration with suppliers, and regulatory pressure) and the dependent variable (adoption of sustainable fuel and renewable energy in logistics), represented as H1-H3. The second layer of relationship tests whether the possible direct relationships are being mediated by organizational commitment, represented as H4a, H4b, and H4c.

## 4. Research hypotheses

### 4.1. Customers’ demand for sustainability and adoption of sustainable fuels and renewable energy in logistics companies

The attention of empirical studies across the globe had been directed towards the importance for firms to be environmentally sustainable [36–38]. This move has been shaped by the increased demand from consumers for firms to enhance their sustainability, both in the environmental and social aspects [39]. Moreover, digital disruption and the recent COVID-19 pandemic increased the need for firms to adopt sustainability approaches in their business processes [40]. Essentially, consumers tend to value green logistics approaches due to the positive influence they have on the environment. Chiamogu and Okoye [41] stated that green logistics is all about taking necessary measures to integrate ecological and environmental issues into supply chain management. It is focused on managing the resources, goods, raw materials, and services of a firm in an effective way that can increase production efficiency, competitive edge, customer satisfaction, and overall performance of the company [42]. Therefore, green logistics strategies aim to avail goods and services in a way that is both economical and beneficial to the ecosystem. They entail waste reduction, preservation of resources, increasing efficiency, and meeting the requirements of society for ecological protection. On the same note, green logistics influences how sustainability is defined in terms of environment, economy, and society, and this is why it is considered a socially and environmentally responsible way of producing and distributing goods and services [43]. Studies have also looked at how customers’ demand for sustainability measures (green logistics) influences the decision of logistics firms to stop adopting sustainability measures, with findings showing that in order to reduce waste, lessen the impact of their operations on human lives and the environment, and conserve energy and resources, these logistics firms need to ensure that eco-friendly practices (like the use of sustainable fuel and eco-friendly energy) are part of the policies and methods in the operations of their suppliers [44]. Overall, consumers now demand firms to adopt green logistics approached through their clear loyalty and interest towards companies that adopt green logistics measures [41]. In view of the above, it is hypothesized that:

Hypothesis 1 (H1): *Customers’ demand for sustainability positively influences logistics companies’ adoption of sustainable fuels and renewable energy.*

### 4.2. Collaboration with suppliers who prioritize sustainability and adoption of sustainable fuels and renewable energy in logistics companies

In considering supply partners’ demand for sustainability and firms’ decision to adopt sustainable fuels and renewable energy in logistics, studies have shown that the movement of goods, processing of information, handling of materials, and exchange of information with supply chain partners are the few examples of the myriads of activities covered by green logistics that influence sustainability in the logistics functions [45]. Nwaulune et al. [46] went further to state that these practices bring about reduction in the environmental issues associated with logistics operations, which include accidents, noise pollution, and greenhouse gas emissions. Through green logistics practices, companies will be able to attain environmental sustainability while also improving their financial performance [47]. On the same note, such practices should encourage the use of reverse logistics, eco-friendly packaging and design, eco-friendly transportation and distribution, eco-friendly procurement, carbon management, eco-friendly warehousing, and other activities, as such will ensure that the supply chain is managed in a manner that is both sustainable and environmentally friendly. In view of this understanding, it is hypothesised that:

Hypothesis 2 (H2): *Collaboration with suppliers who prioritize sustainability significantly enhances the adoption of sustainable fuels and renewable energy in logistics.*

### 4.3. Regulatory pressures and adoption of sustainable fuels and renewable energy in logistics companies

The importance of regulation on the decision of logistics companies to adopt green logistics approaches has also been recorded. In a recent study, Maji et al. [48] assessed the pivotal role played by regulatory frameworks in driving the adoption of clean logistics technologies and making sure that companies create a friendly environment in Bauchi State, Nigeria. Findings from their study showed that an increase in regulatory role increased the adoption of clean logistics technologies, specifically the use of alternative energies. Furthermore, it was also revealed that the adoption of these clean logistics technologies reduced pollution and other environmental issues [48]. This led the authors to recommend that a comprehensive regulatory framework that is founded on the adoption of sustainable practices and the deployment of innovative technologies is pivotal for addressing the challenges that the logistics sector poses to the environment and fostering a global trade ecosystem that is more sustainable [49]. Other empirical studies have analysed the internal and external factors that affect adoption of green logistics practices. In the work of Zimon et al. [50], the author organised the external factors into two more groups: a) consumers/suppliers and b) third parties (such as regulatory pressure, institutional pressure, competition, international environmental regulation, and social responsibility). Regulations and government policies are considered dominant factors [12,16–18,51] that influence adoption of green logistics practices. This view was upheld by Baah et al. [42], as they identified regulatory and organisational stakeholders as vital factors that affect the decision of logistics companies to adopt green logistics practices, which improves the financial performance of the company and environmental reputation. Vienažindienė et al [5] conducted a systematic and comparative analysis of green logistics practice framework for sustainable development, using evidence from the Lithuanian transportation and logistics companies. Findings from the study revealed that the factors that mostly influence adoption of green logistics practices in the companies studied were legal regulations and policies, service users, requirements of business partners, customers, and society, corporate culture that is focused on environmental conservation and sustainable development, and awareness of the company’s top management. Based on these discussions, it is hypothesised that:

Hypothesis 3 (H3): *Regulatory pressure positively affects the adoption of sustainable fuels and renewable energy in logistics.*

### 4.4. Mediating role of organizational commitment to sustainability

A number of the studies have looked at how organisational commitment to sustainability influences adoption of green logistics practices. Jansson [52] stated that the commitment of an organisation to sustainability is about the practices, processes, policies, strategic planning, management philosophies, and competitiveness of an organization. Therefore, the sustainability initiatives adopted and implemented by organisations largely depend on their overall commitment to sustainability because leaders play a significant role in that regard [53–54]. In a more recent study, Shaikh et al. [55] investigated the efforts and role of technology leaders in attaining organisational commitment through the mediating role of green human resource management practices and moderating role of green knowledge sharing. Findings from the study confirm that leadership style does have significant influence on organisational commitment to sustainability, and this influence is mediated by HRM practices and moderated by green knowledge-sharing behaviour. Therefore, organisational commitment, mainly expressed through its leadership style, influences adoption of sustainability policies and practices.

On the same note, corporate leaders play a significant role in uplifting the commitment of employees towards utilising resources that ensure sustainability [56]. Strategic Direction [57] reviewed the latest management developments across the glove and pinpointed practical implications from cutting-edge research and case studies. The study revealed that companies can indicate their genuine commitment to social, economic, and environmental dimensions of sustainability by substantively engaging with their entire stakeholders. It was also revealed that making stakeholder engagement an inbuilt aspect of a company’s voluntary sustainability report does signal concern and awareness to relevant sustainability issues, while on the contrary, reports that limit disclosure of information considered more generic do invite assumptions that preservation of the company’s image is the main focus. Therefore, when companies are truly committed to sustainability initiatives, they tend to draw less attention from the stakeholders because the stakeholders trust such companies to deliver positive sustainability outcomes.

The moderating role of organisational commitment to sustainability on the decision of companies to adopt sustainable practices was further affirmed in the upper echelon theory introduced by Child [58] and further developed by Hambrick and Mason [59]. This theory is founded on the view that the choice of the decision maker (corporate leader that is representing the company) in a given organisation yields a significant impact on the corporate outcome (commitment to sustainability) [60]. Therefore, companies, as well as their leaders, are presumed to vary, and whatever they are committed to will determine the corporate outcome. When they are committed to sustainability, the company will implement more sustainability measures and vice versa.

Hypothesis 4 (H4): *Organizational commitment to sustainability mediates the relationship between stakeholder pressure (customers, suppliers, regulators) and the adoption of sustainable fuels and renewable energy.*

## 5. Methodology

### 5.1. Ethical approval and data availability

The Human Ethical Committee of the Department of Marketing, Faculty of Management Sciences, Enugu State University of Science and Technology (ESUT) approved the study with the approval reference number: ESUT/FMS/MKT/ei2024/004. The committee also monitored the data gathering process to ensure that it was in line with established guidelines. Consent approval was obtained from the respondents in a written form. The first page of the questionnaire contained details and purpose of the questionnaire, and the respondents had to sign their consent before proceeding with the data gathering in the second page. Data was gathered from the 1^st^ of July 2024 to 13^th^ of August 2024. The data gathered and analysed in this research is available at Kaggle (10.34740/kaggle/dsv/6781664)

### 5.2. Sample and data collection

The sample frame for this study includes employees of logistics firms from Nigeria. To assess the assumptions, the researcher surveyed 294 employees of logistics firms from 3 states (Abuja, Lagos, and Enugu) in Nigeria. The selection of these states was done to ensure that employees from the three geopolitical zones (North, West, and East) were represented. This frame was chosen for two main reasons. First, employees of a company represent the most vital source of information when considering adoption of green logistics in a given company, as documented in other related studies [5,13–18]. Secondly, choosing companies from the three geopolitical zones was vital to ensure fair representation of business activities in Nigeria, as focussing on one area might lead to the construing of findings that could poorly represent outcomes from the Nigerian logistics sector.

Furthermore, in a given organisation, the middle management employees represent those that are actively involved in the implementation of green practices and policies, described as “acting leaders.” Therefore, the data for this study was gathered from the middle management employees (of the selected logistics firms) that are actively involved in the process of green practices to produce the desired outcomes (adoption of sustainable fuel and renewable energy) based on stakeholder theory. The researchers made sure that the respondents were employed on a full-time basis and had been working with the company for a certain period of time; as such, it is pertinent to state that these employees are (considerably) acquainted with their company’s commitment to sustainability as well as how the views of stakeholders help influence adoption of sustainability initiatives. A purposeful sampling technique was used to select the respondents for this research. Once the survey questionnaire was selected, a Google form-based web link was generated and sent through WhatsApp Messenger to the selected employees. The researchers were able to access the contact details of the selected employees through the HR of their respective companies. Therefore, consent of the companies was sought to allow their staff to participate in the study.

The online survey link also included a form ensuring the anonymity and confidentiality of the participants. Two separate waves were used to gather the data for this research, and each wave was separated by a 2-week interval in order to help with mitigating possible common method bias and dividing its length. The first survey assessed the views of the employees on the influence of stakeholder theory on the decision of companies to adopt sustainable fuel and renewable energy, while the second survey gathered data on the moderating role of organisational commitment to sustainability. In order to compare the results gathered from each of the two pools, the respondents were issued a special code that they had to enter into the survey. To improve on transparency, these personal codes were further explained to the participants. Necessary measures were also taken to ensure that the rights of respondents were preserved while also preventing social desirability bias (Spector, 2006). On the same vein, the introductory part of the survey stressed that participation was entirely voluntary and all responses would be accorded strict confidentiality.

Following permissions from the human resource departments of the chosen companies, the selected employees were contacted. A total of 680 surveys were sent out, with 359 responses gathered from the first round and another 321 responses gathered from the second round. The final sample consisted of the matching replies from 294 respondents, representing 43.23% response rate.

It has been recommended in PLS-SEM literature that power analyses should be used to determine sample size [61]. However, the decision to adopt power analyses using sample size should be based on the constructs in the model [61]. This view aligns with that of Cohen [62] and other researchers that have recommended the use of statistical power analysis models when computing multiple regressions. The following factors were used to determine the sample size assurance: 1) 80% of statistical power, 2) the lowest value of R-square, and 3) the unpredictability of the model path. Therefore, the final number of responses, 294, carried on for further analyses, was higher than the minimum required sample size of 164. On the same note, the total number of gathered responses was higher than earlier studies [55,63,64] conducted within the same context.

### 5.3. Survey measures

The measurement instruments used in this study were obtained from earlier studies (see Table 1). A scale validity test was conducted for these variables, and the values obtained are: 0.870 for customers’ demand, 0.973 for collaboration with suppliers, 0.889 for regulatory pressure, 0.952 for adoption of sustainable fuel and renewable energy, and 0.949 for organisational commitment. The acceptability criterion for validity scales is 0.7 [65,66], which implies that the scale values obtained in this research are acceptable.

**Table 1:** Survey measure.

Variables	Items	Type	Source
Stakeholder theory	20	IV	[33,35]
Organizational commitment	5	Mediator	[55]
Adoption of sustainable fuel and renewable energy in logistics	7	DV	[63–64]

Source: Authors (2024)

## 6. Results

### 6.1. Data analysis

In order to ensure reliability, validity, and relevance of the structural model linkages, the researchers employed the SPSS statistical analysis program (Version 27) and the SmartPLS software (Version 4.1.0.8) [55]. This approach has gained extensive appraisal for its relevance and usefulness in quantitative research [65–69]. In view of the guidelines provided from the above extant studies, this approach is considered suitable for the quantitative assessment.

### 6.2. Respondents’ demographic profile

This study gathered data from 294 respondents employed across different logistics companies in Nigeria. The demographic profile of respondents is documented in Table 2. In terms of gender, 60% were male and 40% were female. The age shows that the majority were aged 31-35 years old (20%), followed by those aged 36-40 years old (18%), those aged 25-30 years old and above 45 years old (17%, respectively), those aged 41-45 years old (15%), and finally those aged below 25 years old (13%). In terms of the employment status, 100% of the respondents were employed full-time, and this was expected because it was the criterion for participation. Finally, the years of experience show that the majority of the respondents have been in the logistics sector for 5–10 years (53.1), followed by those that have been employed for 1.5 years (37.8%), and finally above 10 years (9.1%).

**Table 2:** Demographic profile of respondents.

Categories	Items	Frequency	Percentage (%)
Gender	Male	176	60.0
	Female	118	40.0
Age	Below 25	38	13.0
	25-30	50	17.0
	31-35	59	20.0
	36-40	53	18.0
	41-45	44	15.0
	Above 45	50	17.0
Employment Status	Full-Time	294	100.0
	Part-Time	0	0.0
	Contract	0	0.0
Years of Experience	1-5 years	111	37.8
	5-10 years	156	53.1
	Above 10 years	27	9.1

Source: Authors (2024)

### 6.3. Measurement model results

For this research, convergent validity, indicator reliability, and composite reliability of the latent constructs were used to confirm the acceptability of the gathered data. Table 3 shows that the values obtained from the loadings show that all the items had 0.6 or above [70,71], indicating validity. Additionally, average variance extracted returned values ≥ 0.5 [72]. Finally, the values for the composite reliability were all higher than the 0.70 threshold, indicating that they met the criteria [66].

**Table 3:** Loadings, composite reliability and average variance extracted.

Latent Constructs	Loadings	Composite Reliability	Average Variance Extracted
Customers’ Demand		0.977	0.589
CD1	0.823		
CD2	0.706		
CD3	0.738		
CD4	0.746		
CD5	0.725		
Collaboration with Suppliers		0.964	0.599
CS1	0.788		
CS2	0.882		
CS3	0.657		
CS4	0.708		
CS5	0.735		
Regulatory Pressure		0.980	0.656
RP1	0.769		
RP2	0.616		
RP3	0.743		
RP4	0.754		
RP5	0.861		
Organizational Commitment		0.991	0.680
OC1	0.727		
OC2	0.766		
OC3	0.779		
OC4	0.890		
OC5	0.746		
Adoption of Sustainable Fuel and Renewable Energy		0.927	0.634
ASFRE1	0.851		
ASFRE2	0.815		
ASFRE3	0.822		
ASFRE4	0.831		
ASFRE5	0.777		
ASFRE6	0.712		
ASFRE7	0.870		

Source: Authors (2024)

Therefore, the returned values show that the convergent validity, composite reliability, and indicator reliability for the latent constructs in this research all fall within the recommended range. Also taken into account during the analysis of the discriminant validity was the criticism of the model [67]. To do this, the hetrotrait-monotrait ratio (HTMT) was used, and the values were under the range of 0.85 [73,74], as documented in Table 4.

**Table 4:** Discriminant validity.

Hetrotrait-monotrait ratio (HTMT)	Customers’ Demand	Collaboration with Suppliers	Regulator Pressure	Organizational Commitment	Adoption of Sustainable Fuel and Renewable Energy
Customers’ Demand					
Collaboration with Suppliers	0.222				
Regulatory Pressure	0.209	0.659			
Organizational Commitment	0.301	0.645	0.727		
Adoption of Sustainable Fuel and Renewable Energy	0.432	0.547	0.601	0.582	

On the same note, the data were gathered across two different waves with a 2-week interval between each wave in order to provide certain mitigation against the common method variance (CMV). To assess the level of CMV as the final measure for statistical control, the Harman’s single-factor test was conducted, as argued by Podsakoff et al. [75]. Furthermore, the investigation shows that the variance inflation factors (VIFs) results produced were all less than the recommended threshold level of 3.0 [76], an indication that the gathered data has no collinearity issue.

The hypothesised model’s predictive power. To determine the strength of the model, the PLS algorithm analysis was used to calculate the value to R^2^. Table 5 documents the R^2^ values for the constructs, showing that the values for customers’ demand, collaboration with suppliers, regulatory pressure, and adoption of sustainable fuel and renewable energy were 51.6%, 77.0%, 67.4%, and 81.0%, respectively. Following the suggestion of Ab Hamid et al. [77], the predictive alliance was not only evaluated with their R^2^ values, as the researcher also evaluated their blindfolded Q^2^ values, and the obtained values were > 0, indicating that they are all within the accepted range [71].

**Table 5:** Predictive relevance of model.

	R square	F square			Q square		
**1**	**2**	**3**	**SSO**	**SSE**	**Q**^**2**^ **(1.SSE/ SSO)**
Customers’ Demand	0.516	0.436			5369.0	3478.10	0.352
Collaboration with Suppliers	0.770	0.579			3490.0	2381.14	0.318
Regulatory Pressure	0.674	0.343			4881.9	2276.03	0.534
Adoption of Sustainable Fuel and Renewable Energy	0.810	0.088			2227.0	1000.411	0.551

**Fig 2:**
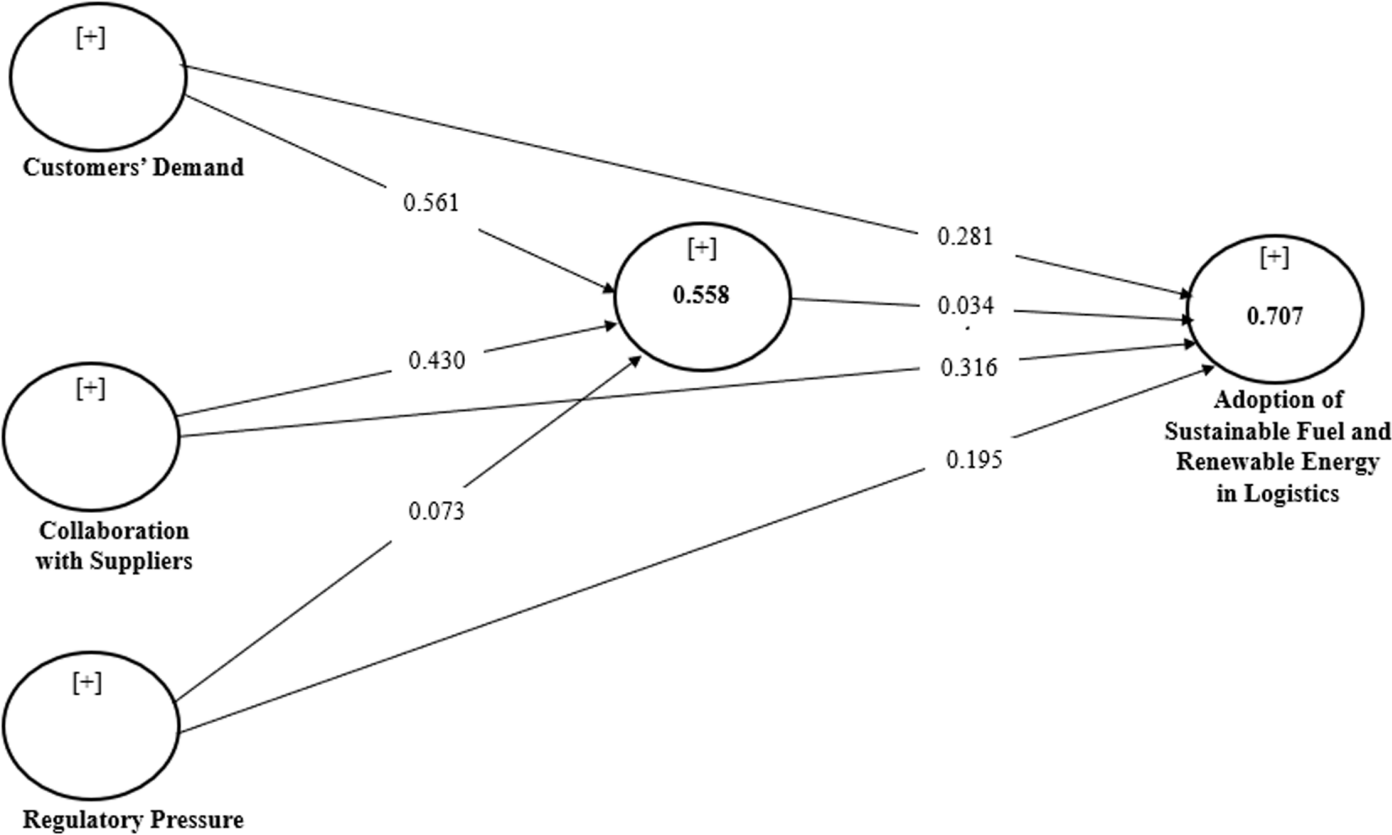
Research model: Path analysis. Source: Authors (2024).

Results of the structural model. To assess the significance of the path coefficient, the researchers used a bootstrapping technique with 5000 bootstrap samples and the 294 observations in line with extant [65–69]. Table 6 reports the coefficients of the structural model analysis, together with the mediator. Table 6 and Fig 2 further validate the association between all the proposed hypotheses.

**Table 6:** Path coefficient.

Hypothesis	Relationship	Beta	SD	*t-*stats	*p* value	Decision
H1	CD → ASFRE	0.281	0.055	5.109	0.000	Supported
H2	CS → ASFRE	0.316	0.021	15.048	0.000	Supported
H3	RP → ASFRE	0.195	0.043	4.535	0.000	Supported
H4a	CD → OC → ASFRE	0.561	0.033	17.000	0.021	Supported
H4b	CD → OC → ASFRE	0.430	0.012	35.833	0.034	Supported
H4c	CD → OC → ASFRE	0.073	0.026	2.808	0.000	Supported

Source: Authors (2024

## 7. Discussion of findings

This paper extensively discussed the contemporary issues associated with adoption of sustainable fuel and renewable energy by logistics companies, a measure employed towards ensuring overall sustainability of the human race. Therefore, it adds to the increasing body of literature on the significant role that logistic companies play in ensuring the sustainability of our ecosystem and the human race. Additionally, it contributes to the literature on organisational commitment by assessing its mediating role in the relationship between stakeholder theory and adoption of sustainable fuel and renewable energy in the logistics sector. Thus, this research augments the literature on stakeholder theory, organisational commitment, and adoption of sustainable fuel and renewable energy in logistics firms within the context of developing economies by providing insights from logistics companies in Nigeria. Little is known about the influence of stakeholder theory on adoption of sustainable fuel and renewable energy in the logistics sector, as well as the mediating role of organisational commitment. Therefore, this research provides significant value by exploring this underexplored area.

The findings from this research are considered significant because all four hypotheses were supported. Considering the significant influence that consumers’ demands play on corporate decisions, it was revealed in this study that consumers’ demands yield positive influence on the decision of logistics firms to adopt sustainable fuel and renewable energy [41,42]

This is mainly based on the fact that companies function to satisfy needs, and their overall performance depends on their ability to satisfy the said needs. Therefore, when the interests of the consumers are not aligned with their corporate values, mission, and objectives, it can potentially lead to a negative effect on their performance. To avert such, they usually tend to pay close attention to the needs of the customers, and such is also applicable with respect to logistic companies’ decision to adopt sustainable fuel and renewable energy in their business processes. Therefore, the finding made in relation to Hypothesis 1 is as expected because the overall sustainability of any company depends on its ability to meet customers’ needs, and the logistics sector is no exception.

Hypothesis (2) focused on collaboration with suppliers. Generally, suppliers have a direct impact on the performance of a company because the quality and consistency of their supply determine the value they can deliver through their final products or services [45–46]. On the same vein, this study revealed that suppliers’ collaboration positively influences adoption of sustainable fuel and renewable energy in logistics firms because when logistics firms are supported by their suppliers to enforce and attain set sustainability goals, the possibility of reaching the said goal increases.

The study also assessed regulatory pressure as a determinant for adoption of sustainable fuel and renewable energy in logistics companies, Hypothesis 3, and among all three elements of stakeholder theory, it was the most researched in relation to sustainability. Similarly, it was revealed that regulatory pressure significantly influences adoption of sustainable fuel and renewable in logistics firms (12, 16-18, 51, 48), and this is expected because regulatory pressure comes with consequences for those that fail to abide by its provisions. Therefore, logistics companies are generally expected to abide by the extant rules and regulations surrounding the adoption of sustainable fuel and renewable energy in their operations.

Finally, the research supported Hypothesis 4, on the ground that organisational commitment mediates the influence of stakeholder theory on adoption of sustainable fuel and renewable energy by logistics firms. This view is also supported by extant studies [53–54]. The major backing force for this hypothesis is that whether policy a company will adopt will significantly depend on the company’s commitment to the said policy. Therefore, it is expected that the extent of influence that the stakeholder theory can yield on the decision of logistics companies to adopt sustainable fuel and renewable energy will depend on the company’s commitment to such influence.

### 7.1. Theoretical implication

This study has significant theoretical implications for the fields of stakeholder theory, organisational behaviour, and sustainability. The research model developed in this study shows that stakeholder theory and organisational commitment are important for adoption of sustainability practices (sustainable fuel and renewable energy) in the logistics sector. The findings from the study align with the relevant theoretical perspectives, the stakeholder theory. As posited in the stakeholder theory, the stakeholders of a company (consumers, suppliers, government, etc.) significantly influence the company’s behaviour and attitude towards certain policies. Therefore, a logistics company’s decision to adopt and implement sustainable policies significantly depends on the stakeholders calls and support for such policies. Therefore, the stakeholder theory suggests building strong relationships between the stakeholders and corporate management as a necessity for sustainable performance, and this is in line with the findings from this research.

### 7.2. Practical and policy implications

The findings from this study have significant practical implications for the stakeholders and companies in the logistics sector. On the side of the stakeholders, it sends a clear signal that they need to apply pressure on logistics companies to adopt sustainability measures in their operational processes because these companies will likely listen once the pressure is coming from the stakeholders. Therefore, stakeholders that are interested in seeing companies in the logistics sector adopt sustainability measures in their corporate processes need to make their desires known through action.

On the side of logistics companies, it also shows that there is a need for them to adopt sustainability measures in the operational processes, especially when there are calls and support for such from the stakeholders. Such sustainable measures include changing from conventional energy resources to sustainable energy and renewable fuels. This can be done by redesigning their corporate process to tailor it with the requirements for sustainable operations, like installing solar panels and so on. This is because the findings have shown that when companies are able to listen to the calls of stakeholders and integrate such into their corporate processes, they are more likely to progress because the stakeholders will reciprocate with loyalty that will help the company remain sustainable.

For the policy makers, in Nigeria, the finding shows that meeting energy emission goals will depend on overall reduction in consumption of conventional energy resources. Therefore, it calls on the policy makers to adopt necessary policies to improve integration, adoption, and penetration of sustainable energy and renewable fuel in the logistics sector and other sectors of the Nigerian economy. Successful development and implementation of such policy is expected to help Nigeria attain its emission goals while create a sustainable future for the next generations.

## 8. Conclusion, limitation and direction for future research

The need to create sustainability through corporate processes is clear, especially with recent global events (such as climate change, flooding, etc.) that tend to question the overall existence of the human race. The logistics sector, just like every other sector, has a significant role to play for the common good of the human race. Evidence from this research has shown that stakeholders can influence the extent to which logistics companies implement sustainability practices by making demands for such to be part of their operational process. On the same note, the companies in this sector should also pay attention to the requests of their stakeholders to ensure alignment towards the central objective of creating a sustainable future for the human race. However, the logistics sector cannot achieve the global sustainability goal alone, and this calls for companies from other sectors to apply similar measures.

While the central objectives of this research have been attained, there are also limitations. However, the limitations in this case do not impact the overall value of the paper; instead, they provide guidelines for further related studies. The first limitation is within the scope of the study. While there are numerous stakeholders (as detailed in the stakeholder theory), this research focused only on the customers, suppliers, and regulatory bodies. Therefore, it is recommended that future studies be conducted to understand the influence of other stakeholders on the decision of logistics companies to adopt sustainability measures. Additionally, this study selected companies from only three states in Nigeria. It is suggested that studying more states from Nigeria or replicating this study in other West African countries can help solidify the application of the findings, at least within the context of the West African region. Finally, this paper recommended adopting more factors as mediating and moderating variables to further strengthen the findings from this research.
